# The Effects of Pitavastatin on Nuclear Factor-Kappa B and ICAM-1 in Human Saphenous Vein Graft Endothelial Culture

**DOI:** 10.1155/2019/2549432

**Published:** 2019-05-02

**Authors:** Bulent Demir, Burak Onal, Sibel Ozyazgan, Esra Demir, Vedat Bakuy, Ahmet Gokhan Akkan

**Affiliations:** ^1^Health Sciences University, Bakırköy Dr. Sadi Konuk Education And Research Hospital, Department Of Cardiology, Istanbul, Turkey; ^2^Biruni Univesity Medical Faculty, Department Of Medical Pharmacology, Istanbul, Turkey; ^3^Istabul Cerrahpasa University Medical Faculty, Department Of Medical Pharmacology, Istanbul, Turkey; ^4^Health Sciences University, Kanuni Sultan Suleyman Education And Research Hospital, Department Of Internal Medicine, Istanbul, Turkey; ^5^Istanbul Baskent University Medical Faculty, Department Of Cardiovascular Surgery, Istanbul, Turkey

## Abstract

**Objective:**

To study pitavastatin's effects on nuclear factor-kappa B (NF-*κ*B ) and adhesion molecules in human saphenous vein graft endothelial culture indicating its pleotropic properties.

**Materials and Method:**

Low-dose (0.1 *μ*M/L) and high-dose (1*μ*M/L) pitavastatin calcium were administered as a frontline therapy in human saphenous endothelial cell culture, followed by induction of inflammation by TNF-*α* and determination of mRNA level alterations of ICAM-1 and NF-*κ*B genes of endothelial cells using the qRT-PCR method. Additionally, immunofluorescence method was used to show the expression of NF-*κ*B and ICAM-1. Finally, LDH levels were determined by the ELISA method to quantify cytotoxicity.

**Results:**

ICAM-1 mRNA expression in the low-dose pitavastatin+TNF-*α* group was significantly higher than that in the TNF-*α* group and significantly lower than that in the high-dose pitavastatin+TNF-*α* group (for all comparisons, P = 0.001). The low-dose pitavastatin+TNF-*α* group had a similar NF-*κ*B mRNA expression with TNF-*α* and high-dose pitavastatin*+*TNF-*α* groups.

**Conclusion:**

Pitavastatin increases ICAM-1 mRNA expression in saphenous vein endothelial cells. Furthermore, the effect of pitavastatin on adhesion molecules appears independent of NF-*κ*B. Novel studies are needed in this field.

## 1. Introduction

In the etiology of atherosclerosis, multiple factors are known to play a role and the pathogenesis of atherosclerosis is known to be extremely complex [1]. As an underlying cause, inflammation plays a predominant role in this complex pathogenesis of atherosclerosis; inflammation takes place not only in the initiation and the progression of atherosclerosis, but also in the development of atherosclerotic complications as acute coronary syndrome [2, 3]. On the other hand, with the huge number of the pathogenesis of atherosclerotic researches at the molecular level, many novel molecular candidates have been introduced as they play part in the atherosclerotic processes. Adhesion molecules have been one of these distinctive candidates with a variety of subtypes [4–7]. These adhesion molecules, intracellular or intercellular adhesion molecule-1 (ICAM-1) or CD54 (Cluster of Differentiation 54), undertake important functions. ICAM-1, a member of the immunoglobulin superfamily, found in the transmembrane glycoprotein structure is being expressed in many tissues such as fibroblasts and gastrointestinal system tissues especially in endothelial or epithelial cells, macrophages, monocytes, and T and B lymphocytes [7, 8]. ICAM-1's role in the circulation is to facilitate attachment of leukocytes to endothelial cells and help the transendothelial passage and migration of leukocytes to the site of inflammation [7, 9]. Thus, ICAM-1 seems to play an important role in the development of atherosclerosis, thrombosis, and inflammation [9, 10]. Nuclear factor-kappa B (NF-*κ*B) is an important transcription factor that has been found to have many functions over the last 30 years [11]. NF-*κ*B also plays an important role in pathogenesis of atherosclerosis and its expression has been shown in atherosclerotic plaques [11, 12]. NF-*κ*B is especially found to be critically important in controlling the genes of molecules involved in many stages of atherosclerosis, such as cytokines, chemokines, adhesion molecules, and acute phase reactants [13]. In response to inflammatory stimulation, NF-*κ*B shows regulatory functions in the expression of adhesion molecules, including ICAM- 1 and VCAM-1 [13]. Studies have shown that ICAM-1 and VCAM-1 transcription are largely dependent on the induction of tumor necrosis factor-*α* (TNF-*α*) through the NF-*κ*B activation [14, 15]. Therefore, NF-*κ*B and adhesion molecules that play a role in the progression of atherosclerosis are expected to be in a close relationship. Pitavastatin the recent clinically approved HMG-CoA is a reductase inhibitor [16]. In the recent studies, pitavastatin was shown to have many pleiotropic effects [16]. One of the most important pleiotropic effects of pitavastatin is its anti-inflammatory effect. Vascular inflammation is known to be at the center of atherosclerosis and, in this context, anti-inflammatory effects become important as it was shown in the cerebral aneurysm models in rats that pitavastatin managed to reduce NF-*κ*B activation [16, 17]. Currently, a significant factor affecting the success of the coronary artery bypass graft (CABG) treatment is found to be the retention of saphenous vein graft causing saphenous disease and its related graft occlusions. Thus in the treatment of saphenous graft disease, application of cholesterol-lowering statins as treatment gains importance.

In the recent studies on the patient who had CABG with saphenous vein harvestmen, the usage of statins led to a better reduction on LDL and an extra time to remain open. In addition to that, besides the lipid-reducing properties of statins, data showing the improvement of the endothelial function, reducing vascular inflammation and oxidative stress, and improving saphenous vein graft disease were also presented. However, the effect of pitavastatin on the adhesion molecules in human saphenous vein graft endothelial cells is yet not clear. In the pathogenesis of saphenous graft disease, adhesion molecules such as NF-*κ*B and ICAM-1 together with VCAM-1 appear to be on the inflammation pathway. That is why in our study we aimed to investigate the effects of pitavastatin on NF-*κ*B and the important ICAM-1 expression on the endothelial cell culture being induced by daily used TNF-*α* and inflammation.

## 2. Material and Method

This endothelial culture study, PromoCell C-12231, was carried on human saphenous vein endothelium. The endothelial cells were provided and were replicated after being dissolved according to the protocol. The steps of our study as the preparation of saphenous vein endothelial cell cultures and the stimulation of endothelial cells by TNF-*α* to form the inflammation model, followed by pitavastatin application, the isolation, and dying of the mRNA for the endothelial cells were performed in the Neurodegenerative Diseases Research Laboratory. cDNA synthesis, determination of LDH levels by ELISA, and gene expression assays were performed in Pharmacogenetic Research Laboratory of Medical Pharmacology Department of Our Medical Faculty.

## 3. Human Saphenous Vein Endothelial Cell Experiment

Human saphenous vein endothelial cells used in our study were supplied with the catalog number of Promo Cell C-12231 (Promocell Heidelberg, Germany). Pretreatment was performed to all the experimental groups prior to the experimental procedure applied in the saphenous vein endothelial cell culture. Pitavastatin (Santa Cruz Biotech SC-208176A) was applied to all endothelial cell culture groups for an hour. Procedure was followed by TNF-*α* (Life Tech. PHC3011, Carlsbad, CA, 92008, USA) pitavastatin doses and experimental groups were formed as follows:

1. Control group: endothelial cells without any type of treatment was defined as the untreated control group
2. Low-dose pitavastatin group (PTV1): endothelial culture cells with 0.1 *μ*M/L of pitavastatin calcium application for an hour were defined as the low-dose pitavastatin group.
3. High-dose pitavastatin group (PTV2): endothelial culture cells with 1 *μ*M/L of pitavastatin calcium application for an hour were defined as high-dose pitavastatin group.
4. TNF-*α* group: endothelial culture cells with solely 10 ng/mL of TNF-*α* application for six hours.
5. Low-dose pitavastatin + TNF-*α* group (PTV1 + TNF-*α*): endothelial culture cells with prior 0.1 *μ*M pitavastatin calcium application for an hour followed by TNF-*α* administration for 6 hours.
6. High-dose pitavastatin + TNF-*α* group (PTV2 + TNF-*α*): endothelial culture cells with prior 1 *μ*M of pitavastatin calcium application for an hour followed by TNF-*α* administration for 6 hours.
7. DMSO group: defined as the group pitavastatin, calcium, and TNF-*α* was dissolved in 5*μ*M of DMSO *∗*

For DMSO application, the highest level of DSMO in PTV2 group was taken into consideration and the concentration was prepared as 5*μ*M; thus the highest doses' cytotoxicity was calculated and, for any lower doses, no extra groups were formed.

## 4. Application of Immunofluorescence Staining Protocol for NF-* *-**κ**B and ICAM-1 Proteins

In order to perform immunofluorescence staining of NF-*κ*B proteins with ICAM-1, a dual staining procedure was planned.

Firstly, the cultured endothelial cells were fixed in a 3,7% paraformaldehyde (Sigma 15,812-7. Sigma-Alderich Chemie GmbH, Steinheim, GE) for 30 minutes. After fixation, endothelial cells were washed 5 times with 2000 *μ*l PBS for 10 minutes. After the bathing stage, endothelial cells in 0.01% normal goat serum (Chemicon S26. Millipore Corp., California, USA) were prepared in PBS containing 0.01% Triton X-100 (Sigma X-100. Sigma-Alderich Chemie GmbH, Steinheim, GE) and were incubated for an hour. After incubation, the endothelial cells were reconstituted with Anti-ICAM-1 (Abcam-ab20144, Cambridge, UK) primary antibody and 30% goat serum prepared in 0.01% T-PBS with a dilution of 1:50 and left overnight at + 4°C. At this stage, 30% goat serum prepared in 0.01% T-PBS without primary antibody was applied to the negative controls and the first day of the staining procedure was completed.

On the second day of the staining procedure, the cultured endothelial cells were washed 5 times with 2000 *μ*l of PBS for 10 minutes. Then 1.5% goat serum in 0.01% T-PBS with goat anti-mouse IgG, H & L (Alexa Fluor 488) (Abcam-ab150177, Cambridge, UK) was diluted in secondary antibody as 1: 100 in a dark medium was incubated for an hour at room temperature. After incubation steps, endothelial cells were washed 3 times in dark medium at room temperature for 10 minutes with 2 ml PBS.

In the next stage, endothelial cells were kept at room temperature for 30 minutes in a 10% normal goat serum that was prepared in 0.01% T-PBS. The endothelial cells were then reconstituted with primary antibody NF-*κ*B (Abcam-ab32536, Cambridge, UK) in a ratio of 1: 100 with 10% goat serum prepared in 0.01% T-PBS and were incubated for 2 hours. 10% goat serum without primary antibody was administered. After two hours of incubation, endothelial cells with goat anti-rabbit IgG Alexa Fluor 568 (Red) (Abcam-ab150079, Cambridge, UK) secondary antibody diluted 1: 100% in 0.01% T-PBS in 1.5% goat serum was incubated for an hour. Then the process of closing the knitting of the dying process was done using the closing medium.

In the next step, the endothelial cells were examined in an inverted fluorescence microscope followed by the photo shooting by fluorescent camera system using the TR, I3, and A3 filters (Leica DFC 300 FX, Leica Microsystems Ltd., Heerbrugg, GE, etc.). After photos were taken, the matching of the photographs of the same area with different filters and using the special software (Leica Application Suite Image Overlay Software, Leica Microsystems Ltd., Heerbrugg, GE) was completed. Then, the pixel density of 10 photographs taken from randomly determined regions of each petroleum was determined using Image J software.

In the last stage, the numerical data of each photograph were determined separately. Thus, statistical analysis of numerical data was obtained and the differences between the pixel intensities reflecting the expression of ICAM-1 and NF-presyonB proteins were determined.

## 5. Isolation of RNA from Endothelial Cells

RNA isolation of human saphenous vein endothelial culturing cells was performed according to the protocol of RNA isolation kit (Life Tech AM1728 Cells-To-CT). After cDNA synthesis was complete, by qRT-PCR method, the amounts of mRNA fluctuation were determined. The qRT-PCR method is available with the Applied Biosystems 7500 Fast (Life Tech. C arlsbad, CA, 92008, USA), TaqMan Probes, and FG TaqMan Cells-To-CT (AM1728, Life Tech., Carlsbad, CA, 92008, USA) kit.

-TaqMan Gene Expression Test for VCAM-1 Human VCAM-1 Hs01003372_m1 - 4331182 Life Tech., Carlsbad, CA, 92008, USA.

For ICAM-1, -TaqMan Gene Expression Test Human ICAM-1 Hs00164932_m1 - 4331182 Life Tech., Carlsbad, CA, 92008, USA.

For NF-*κ*B, - TaqMan Gene Expression Assay Human NF-7B Hs00765730_m1 - 4331182 Life Technology, Carlsbad, CA, 92008, USA.

-TaqMan Gene Expression Test Human ACBT Hs01060665_g1 - 4331182 Life Tech., Carlsbad, CA, 92008, United States.

400 *μ*l of the sample to determine the level of LDH for ELISA was taken and was maintained at -80°C until the day of study. To measure the LDH amount being released to the media, kit of Cytotoxicity Detection (LDH) (Roche 11 644 793 001, Roche Diagnostics GmbH Roche Applied Science Mannheim, GE) was used.

## 6. Statistical Analysis

The data obtained from the study were analyzed with SPSS software version 22.0. Mean, median, lowest, highest, and standard deviation values were used in the descriptive statistics.

The distribution of the data was tested by one-sample Kolmogorov-Smirnov test. And the data did not show a normal distribution; Kruskal-Wallis test was used with Mann-Whitney* U* test. Statistical significance was accepted as p <0.05.

## 7. Results

The ICAM-1 relative mRNA expression levels of the experimental groups were summarized in Table 1.

NF-*κ*B relative mRNA expression levels of the experimental groups were summarized in Table 2.

ICAM-1 Pixel Density Results of the Experimental Groups summarized in Table 3. NF-*κ*B Pixel Density Results of the experimental groups are summarized in Table 4.

## 8. Comparison of ICAM-1 mRNA Expression Levels between Groups

Comparison results of mRNA expression for ICAM-1 levels between groups are summarized in Table 5. In terms of ICAM-1 mRNA Expression, there was no statistically significant difference between the control group and low-dose pitavastatin and high-dose pitavastatin (P = 0.398, P = 0.432, respectively ) (Table 5). However, the level of ICAM-1 mRNA expression in the control group; was significantly lower than in the TNF-*α*, low-dose pitavastatin + TNF-*α*, high-dose pitavastatin + TNF-*α* and DMSO groups (for all P = 0.001) (Table 5).

**Figure 1:**
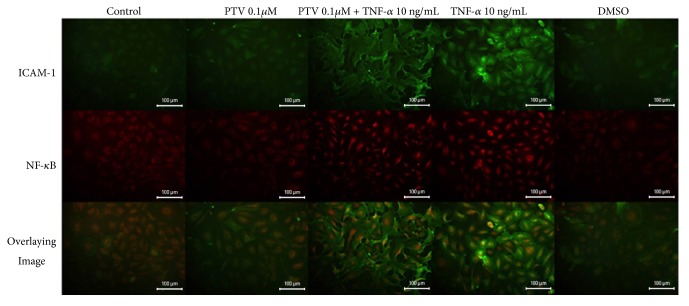
Immunofluorescent staining images of ICAM-1 and NF-***κ***B in the endothelial culture treated with 0.1 *μ*M pitavastatin and the conclusions of two images. 20X magnification. ICAM-1, intercelluler adhesion molecule-1; NF-*κ*B, nuclear factor-kappa B; PTV1, low-dose pitavastatin (0.1 *μ*M / L); PTV1 + TNF-*α*, low-dose pitavastatin (0.1 *μ*M / L) + tumor necrosis- alpha (10 ng / mL); TNF-*α*, tumor necrosis-alpha (10 ng / mL); DMSO, dimethyl sulfoxide.

When high-dose pitavastatin group was compared with Low-Dose Pitavastatin group, there was no statistically significant difference in terms of mRNA Expression level (P = 1.00) (Table 5) (Figure 1). However, the level of ICAM-1 mRNA expression in the low-dose pitavastatin group and TNF-*α* was significantly lower than Low-Dose Pitavastatin + TNF-*α*, high-dose pitavastatin + TNF-*α*, and DMSO groups (for all P = 0.001) (Table 5).

The mRNA expression level of ICAM-1 in the high-dose pitavastatin group was significantly lower than the mRNA expression level of ICAM-1 in TNF-*α*, Low-Dose Pitavastatin + TNF-*α*, high-dose pitavastatin + TNF-*α*, and DMSO groups (P = 0.002 for all) (Table 5).

On the other hand, in the low-dose pitavastatin + TNF-*α* group, the ICAM-1 mRNA expression level was significantly higher than the TNF-*α* group (P = 0.001) (Table 5) (Figure 1). Similarly, in low-dose pitavastatin + TNF-*α* group, ICAM-1 mRNA expression level was significantly higher than DMSO group (P = 0.001) (Table 5). However, in the low-dose pitavastatin + TNF-*α* group, the level of ICAM-1 mRNA expression was significantly lower than the high-dose pitavastatin + TNF-*α* group (P = 0.001) (Table 5).

And in high-dose pitavastatin + TNF-*α* group the level of ICAM-1 mRNA expression was found to be significantly higher compared to solely TNF-*α* and DMSO groups (P = 0.001 for both) (Table 5).

Lastly, when the DMSO group and the TNF-*α* group were compared the TNF-*α* showed a significantly higher level of ICAM-1 mRNA expression than in DMSO group (P = 0.001).

## 9. Comparison of NF-**Κ**B mRNA Expression Levels between Groups

Comparative tables showing NF-*κ*B mRNA expression between the groups are summarized in Table 6.

When mRNA expression levels of NF-*κ*B are compared control group showed higher levels of mRNA expression than both low and high doses of pitavastatin groups (P = 0.001, P = 0.002, respectively) (Table 6). On the other hand, mRNA expression level of NF-*κ*B was significantly lower in TNF-*α*, Low-Dose Pitavastatin + TNF-*α*, and high-dose pitavastatin + TNF-*α* group (P = 0.001, P = 0.001, P = 0.001, respectively) (Table 6). Furthermore, in terms of mRNA expression, no significant evaluation was established between the control group and the DMSO group (P = 1,000).

On the other hand, in the low-dose pitavastatin group, level of NF-*κ*B mRNA expression was lower than the groups including high-dose pitavastatin, TNF-*α*, Low-Dose Pitavastatin + TNF-*α*, high-dose pitavastatin + TNF-*α*, and DMSO (P = 0.002, P = 0.001, P = 0.001, P = 0.001, and P = 0.001, respectively) (Table 6).

In the high-dose pitavastatin group, mRNA expression level NF-*κ*B was lower compared to TNF-*α*, Low-Dose Pitavastatin + TNF-*α*, high-dose pitavastatin + TNF-*α*, and DMSO groups (P = 0.002, P = 0.002, P = 0.002, and P = 0.002) (Table 6).

In low-dose pitavastatin + TNF-*α* group, the level of NF-*κ*B mRNA expression was significantly not different from the TNF-*α* and high-dose pitavastatin + TNF-*α* groups (P = 0.275, P = 0.673) (Table 6).

But the level of NF-*κ*B mRNA expression in low-dose pitavastatin + TNF-*α*, was significantly higher than in DMSO group (p = 0.001) (Table 6).

High-dose Pitavastatin + TNF-*α* group and TNF-*α* alone showed no significant difference in the term of NF-*κ*B mRNA expression level (P = 0.205) (Table 6).

For TNF-*α* group, the level of NF-*κ*B mRNA expression was higher than in DMSO group (P = 0.001) (Table 6).

## 10. Comparison of ICAM-1 Pixel Density

The control group and the low-dose pitavastatin group showed no significant difference in terms of the intensity of the pixel (P = 0.133) (Table 7).

But the control group's ICAM-1 pixel density was lower compared to high-dose pitavastatin, TNF-*α*, Low-Dose Pitavastatin + TNF-*α*, high-dose pitavastatin + TNF-*α*, and DMSO groups (P = 0.006, P = 0.004, P = 0.001, P = 0.001, and P = 0.032) (Table 7).

Low-dose pitavastatin group and the DMSO group showed no difference in terms of the pixel density of the ICAM-1 (P = 0.565) (Table 7).

ICAM-1, high-dose pitavastatin, TNF-*α*, Low-Dose Pitavastatin + TNF-*α*, and high-dose pitavastatin + TNF-*α* groups were significantly lower and lower (P = 0.028, P = 0.003, P = 0.001, and P = 0.001, respectively) (Table 7).

Even though ICAM-1 pixel density in the high-dose pitavastatin group was significantly lower than TNF-*α*, Low-Dose Pitavastatin + TNF-*α*, and high-dose pitavastatin + TNF-*α* groups (P = 0.006, P = 0.002, and P = 0.003, respectively), the difference between high-dose pitavastatin + TNF-*α* group and DMSO group was not statistically significant (P = 0.123) (Table 7).

Another important result was between the Low-Dose Pitavastatin + TNF-*α* group and TNF-*α* and high-dose pitavastatin + TNF-*α* groups; ICAM-1 pixel density did not show any significant difference (P = 0.386, P = 0.624) (Table 7). But low-dose pitavastatin + TNF-*α* group showed higher ICAM-1 pixel density than DMSO group (P = 0.001) (Table 7).

On the other hand with the high dose, pitavastatin + TNF-*α* group and TNF-*α* group showed no significant difference in terms of ICAM-1 pixel density (P = 0.814) (Table 7). But ICAM-1 pixel density of high-dose pitavastatin + TNF-*α* group was higher than the DMSO group (P = 0.001) (Table 7). Lastly, ICAM-1 pixel density of TNF-*α* group was statistically higher than the DMSO group (P = 0.003) (Table 7).

## 11. Comparison of NF-**Κ**B Pixel Density

In the TNF-*α* group, the pixel density of NF-*κ*B was statistically higher than the control group (P = 0.006) (Table 8). Similarly, NF-*κ*B pixel density in low-Dose Pitavastatin + TNF-*α* group was significantly higher than in control group (P = 0.034) (Table 8). Also, NF-*κ*B pixel density in TNF-*α* group was significantly higher in other groups as low-dose pitavastatin, high-dose pitavastatin + TNF-*α*, and DMSO group (P = 0.004, P = 0.011, P = 0.039, and P = 0.004). The comparison of the groups showed no significant difference in terms of NF-*κ*B pixel density (P> 0.05, respectively) (Table 8).

## 12. Fluorescent Microscopic Evaluation

**Figure 2:**
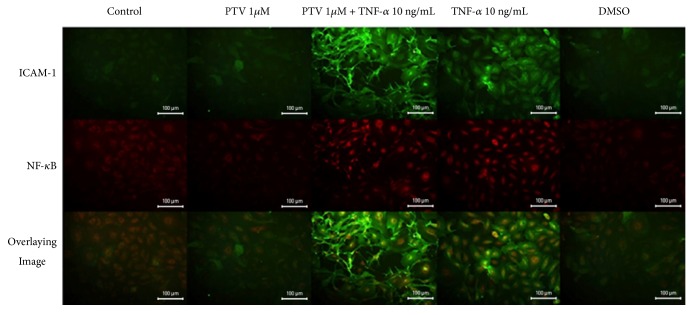
Immunofluorescent staining images of ICAM-1 and NF-***κ***B in the endothelial culture treated with 1 *μ*M pitavastatin and the conclusions of two images. 20X magnification. ICAM-1, intercelluler adhesion molecule-1; NF-*κ*B, nuclear factor-kappa B; PTV1, high-dose pitavastatin (1 *μ*M / L); PTV1 + TNF-*α*, high-dose pitavastatin (1 *μ*M / L) + tumor necrosis-alpha (10 ng / mL); TNF-*α*, tumor necrosis-alpha (10 ng / mL); DMSO, dimethyl sulfoxide.

Immunofluorescent staining for ICAM-1 and NF-*κ*B was applied to the human saphenous vein endothelial culture that previous pitavastatin was applied, followed by TNF-*α* application and 6 hours of incubation. Images of immunofluorescence staining were given in Figures 1 and 2.

## 13. Immunofluorescence Staining Results for ICAM-1

In the control and DMSO groups, weak immune positivity was observed for ICAM-1 in the cells. In the group treated with 0.1*μ*M pitavastatin, weak immunostaining was observed for ICAM-1. In 0.1 *μ*M pitavastatin and TNF-*α* group, a strong immune positive reaction was observed and this immune positivity was significantly localized in the cell membrane. In the group with 1 *μ*M pitavastatin application, weak immune positivity was observed for ICAM-1. The group where pitavastatin and TNF-*α* were applied together showed a very strong immunopositivity compared to the group of control and 1 *μ*M pitavastatin. Similarly, in group where 0.1 *μ*M pitavastatin was applied and the group where 1 *μ*M pitavastatin and TNF-*α* coadministered together were compared with the other groups immune positivity was found to be stronger. A strong ICAM-1 immune positive reaction was observed in the TNF-*α* group.

The endothelial cells in this group showed different appearance from the other groups and showed a diffuse morphology.

## 14. Immunofluorescence Staining Results for NF-**κ**B

In the control group and in the DMSO group, immunoblotting was observed in the cytoplasm and in the nucleus.

NF-*κ*B immunopositivity was decreased slightly in the 0.1 *μ*M pitavastatin treated group compared to the control group.

In 0.1 *μ*M pitavastatin and TNF-*α* coadministrated group, NF-*κ*B immunopositivity was found to be increased compared to the control group and mostly was localized in the nucleus.

NF-*κ*B was found to be weaker than the control group.

In the group with 1 *μ*M pitavastatin and TNF-*α* coadministration, control for NF-*κ*B and only 1 *μ*M were denser and mostly localized in the nucleus, and some in the cytoplasm.

A strong immune positive reaction localized in the nucleus for NF-*κ*B was observed only in the TNF-*α* treated group. There was also a prominent cytoplasmic presence.

## 15. Comparison of LDH Levels

Results of LDH levels of experimental groups are summarized in Table 9. In TNF-*α* group, the LDH level was significantly lower than the high-dose pitavastatin group (P = 0.010) (Table 10). Similarly, LDH level in the low-dose pitavastatin + TNF-*α* group, was significantly lower than the high-dose pitavastatin group (P = 0.025) (Table 10). In addition, LDH levels in the high-dose group of pitavastatin + TNF-*α* were found to be significantly higher than the low-dose pitavastatin + TNF-*α* group (P = 0.016) (Table 10). Similarly, the LDH level in the high-dose pitavastatin + TNF-*α* group was higher than the TNF-*α* group alone (P = 0.010) (Table 10). No statistically significant difference was found in the comparison of LDH levels among the other groups (for all, P> 0.05) (Table 10).

## 16. Discussion

Our study has reached two important results. Firstly in the human saphenous vein grafts endothelium pretreated with pitavastatin and stimulated by TNF-*α* to form inflammation model, expression of ICAM-1 was increased. Secondly, in the inflammation models that were formed by TNF-a stimulation being pretreated with low doses or high doses of pitavastatin, NF-*κ*B expression was not altered significantly. As far as we know our study is the first to examine mRNA expression of ICAM-1 and NF-*κ*B in endothelial cell culture of saphenous vein grafts induced by TNF-a with the treatment of pitavastatin. It is known that saphenous vein grafts are widely used in CABG operation but also show problems due to development of saphenous graft occlusions as saphenous graft disease develops. There are data showing that statin group antihyperlipidemic drugs can be useful in the treatment of saphenous vein graft disease [18]. However, studies on the molecular mechanisms of the effects of statins on adhesion molecules and vascular inflammation are limited. As the statin group, antihyperlipidemic drugs are used in CABG patients in the daily basis; we consider our results especially important while the results might have an impact on clarifying some of the molecular basis of the treatment in use. Even though there are many studies evaluating the effects of statin group antihyperlipidemic drugs on adhesion molecules that play an important role in the development of vascular inflammation and atherosclerosis, not enough data for pitavastatin, the latest HMG-CoA reductase inhibitor are valid. One of the most important pleiotropic effects of statins is the anti-inflammatory effect. However, our study showed that pretreatment pitavastatin was observed to increase ICAM-1 expression as opposed to expected and this finding can be interpreted as an interesting result. Although increase in the expression of TNF-*α* induced ICAM-1 by the pretreatment of pitavastatin is suspected to be an interesting and unexpected result, similar findings in the literature support our results [19, 20].

The potential molecular mechanism of increased expression of TNF-*α*-induced ICAM-1 by statins appears to be the inhibition of generalization which is very important for the function of small G proteins [19] while the addition of mevalonate or geranylgeranyl pyrophosphate (GGPP) reverses the excessive induction of adhesion molecules [19]. By the HUVEC study of Bernot D et al. it is demonstrated that atorvastatin in endothelial cells significantly increased TNF-*α*-induced ICAM-1, VCAM-1, and E-selectin expression compared to the TNF-*α* alone group [19]. Another HUVEC study, conducted with Lovastatin pretreatment, induced ICAM-1, VCAM-1 and E-selectin expression in TNF-*α*-induced endothelial cells in concordance with our results [20]. Another striking result of this study was the revealed with investigation by confocal microscopy, as atorvastatin increases the expression of adhesion molecules in endothelial cells as well as altering the distribution of adhesion molecules on the cell surface [19].

As a result of these findings, atorvastatin was found to reduce the adhesion of monocytes to endothelial cells by about 42% as an effect [19]. Although the increase in ICAM-1 expression, at first sight, might be interpreted as an increase in proinflammatory activity; in reality, the statins exhibit anti-inflammatory and antiatherosclerotic properties by altering the distribution of adhesion molecules on the cell surface or, more importantly, disrupting the function of adhesion molecules. In this context, in the study by Weitz-Schmidt G et al. it is found that LFA-1, the integrin to which ICAM-1 is attached, shows selectively to be inhibited by atorvastatin that blocks the binding site of ICAM-1 [21]. Thus, although increasing effects of pitavastatin on ICAM-1 expression seem to show proinflammatory effect, statins as they inhibit the interaction of ICAM-1 with LFA-1 might ultimately cause anti-inflammatory effects. New studies are needed to clarify the effect of pitavastatin on the interaction between ICAM-1 and LFA-1. On the other hand further studies including the mevalonate or GGPP are needed to elicit the overexpression of ICAM-1 due to inhibition of the synthesis of isoprenoid derivatives such as farnesyl pyrophosphate (FPP) and geranyl pyrophosphate (GGPP), which acts in intermediate steps in cholesterol biosynthesis due to inhibition of mevalonic acid synthesis or not. Also as in our experience with pitavastatin, the distribution of the ICAM-1 adhesion molecule was not studied at the cell surface level and further data is required.

There might be other possible reasons for TNF-*α* induction due to experimental design and its protocols. In our experiment pitavastatin was applied for the short term; it is possible that chronic administration just like in the clinical practice of pitavastatin can alter these results whereas the HMG-CoA reductase inhibitors are lifelong drugs being used. Another possible explanation for the ICAM-1 overinduction is due to the specific molecular structure of the pitavastatin. In this context, Niwa S et al. in one of the HUVEC culture studies showed that fluvastatin did reduce the ICAM-1 expression, whereas another HMG-CoA reductase inhibitor, Pravastatin, did not affect ICAM-1 expression [22]. Therefore, the differences in the cell, tissue, and organ specificity for HMG-CoA reductase inhibitors may explain the negative impact of pitavastatin on ICAM-1 expression [22]. In addition, several pharmacological differences, such as potency of pitavastatin, affinity for HMG-CoA reductase enzyme, and lipophilicity, can be cited as the reasons behind this negative effect on ICAM-1 expression. As there is no study evaluating the effect of pitavastatin on direct ICAM-1 expression, for a better conclusion, new studies are needed to evaluate the effects of various HMG-CoA reductase inhibitors on ICAM-1 expression including pitavastatin. As the third reason the doses of pitavastatin administration in our assay protocol might be insufficient for the inhibition of ICAM-1 expression. Low-dose pitavastatin might increase ICAM-1 expression; pitavastatin doses to be administered at higher doses than we do may reduce ICAM-1 expression. In order to clarify this issue, new studies with higher doses of pitavastatin are needed without exceeding to toxic doses.

As the fourth reason, treatment with pitavastatin may be acutely inducing ICAM-1 expression as in some cases anti-inflammatory molecules can act as proinflammatory. In addition, the inhibition of pitavastatin on ICAM-1 expression may increase the expression of ICAM-1 as a reply to rebound response. As the fifth reason, the anti-inflammatory effect, which is one of the pleiotropic effects of pitavastatin, may not be playing a role in inhibiting the expression of ICAM-1. In endothelial cells, pitavastatin might induce ICAM-1 expression in the acute phase duration and cause a proinflammatory effect. It is also known that there may be negative effects of HMG-CoA reductase inhibitors. In heart failure studies including patients with ischemic heart failure, the positive effects of statins on mortality have not been demonstrated [23]. In particular, the inhibition of ubiquinone synthesis by statins in cardiac myocytes; impairing the mitochondrial function has been described as one of the possible causes of the lack of expected benefit in heart failure [23]. As a result, the most valid theory to explain the mechanisms of overexpression of pitavastatin TNF-*α* induced ICAM-1 is the inhibition of synthesis of isoprenoids due to inhibition of mevalonate synthesis among possible theories and new studies are needed to clarify this issue.

Also, when the density of pixels of immunofluorescence staining of ICAM-1 adhesion protein was evaluated among the groups; administration of TNF-*α* increases the expression of ICAM-1 on the endothelial cell surface, while high-dose pitavastatin pretreatment further enhances the expression of ICAM-1 on the TNF-*α*-induced cell surface. But this increase has not gained statistical significance. This difference might be a consequence of qRT-PCR method in which method for detection of mRNA expression by immunofluorescent staining is based on different foundations. On the other hand, since each mRNA molecule synthesized may also require some postsynthesis modifications, and this process cannot be successfully completed; the mRNA molecule's inability to turn into the target protein may also explain this statistical nonsignificance.

Another important point of our study is that although NF-*κ*B and TNF-*α* expression were significantly induced in saphenous endothelial cells, low- and high-dose pitavastatin pretreatment on NF-*κ*B expression induced by TNF-*α* were not statistically significant. However, immunofluorescent staining of NF-*κ*B pixel density shows that NF-*κ*B protein, which is being expressed as a result of NF-*κ*B induction after being treated with different doses of pitavastatin, is reduced and with the high doses of pitavastatin pretreatment NF-*κ*B pixel density lost its significance. Thus, it may be caused by difference in protocols and expression of every single mRNA molecule does not mean they are going to be transformed into target protein as many posttranscriptional and translational processes are required; additionally, our findings of mRNA expression were relative results. But results suggest that reduction in NF-*κ*B pixel inhibition is because the VCAM-1 expression was inhibited by high-dose pitavastatin pretreatment; so it can be thought that NF-*κ*B plays an active role on VCAM-1 expression.

One of the important studies among the limited studies on NF-*κ*B is a rat model of cerebral aneurysm where pitavastatin was found to inhibit NF-*κ*B expression [17]. Similar to our work in human saphenous vein endothelial cell culture, pitavastatin was found to inhibit apoC III-induced NF-*κ*B activation [24]. In our study, we concluded that pretreatment of pitavastatin did not significantly alter the relative mRNA level when induced by TNF-*α*. The reason might be the complex process of cytokine-induced ICAM-1 expression, where transcription proteins play a role in regulation of adhesion molecules expression besides taking a role in cytokine-induced ICAM-I expression. In this context, a nuclear receptor and Peroxisome proliferator-activated receptor (PPAR-*γ*) are also important in ICAM-1 expression [25].

Wang N et al. demonstrated that activation of PPAR-*γ* in human endothelial cells reduces the expression of adhesion molecules [26]. In addition, ICAM-1 and VCAM-1 expression are known to play a role in activating protein-1 (AP-1), another nuclear transcription factor [27, 28]. As a result, ICAM-1 and VCAM-1 appear to play a role in different nuclear receptors such as NF-*κ*B as well as other nuclear transcription factors and PPAR. Thus, in our study, ICAM-1 expression induced by pitavastatin pretreatment might be not only due to NF-*κ*B, but also due to the effects on different nuclear receptors such as PPAR. To support this, data on PPAR receptors are also available but still more is required [29–31].

Finally, when the LDH levels were measured for the evaluation of the cytotoxicity of pitavastatin applied groups, lower LDH levels were obtained in the group of high-dose pitavastatin compared to low-dose pitavastatin. This suggests that high-dose pitavastatin is more cytoprotective. In the same way, the LDH level was increased in TNF-*α* group because of inflammation induced cytotoxicity. LDH levels were significantly decreased, especially in high-dose pitavastatin pretreatment + TNF-*α* group. This result strongly supports that the high-dose pitavastatin prophylaxis has markedly increased the protective effect of endothelial cells in the environment of inflammation. Thus, it may be concluded that the use of high-dose pitavastatin in the treatment of saphenous vein graft disease and coronary artery disease may be more beneficial.

As a result, especially high-dose pitavastatin may be used in the prevention and treatment of saphenous vein graft disease. However, new experimental studies are needed to clarify the molecular basis of the beneficial effect of pitavastatin in the treatment of saphenous graft disease. On the other hand, in order to evaluate the success of pitavastatin treatment in maintaining saphenous vein graft patency, it is clear that clinical studies are needed to evaluate saphenous graft patency with long-term angiographic follow-up in patients with CABG operation and saphenous vein grafts, because, in some cases, the results obtained in daily clinical practice with experimental results may be different.

## Figures and Tables

**Table 1:** Results of ICAM-1 mRNA expression levels of experimental groups.

EXPERIMENT GROUP NAME	ICAM-1
	Mean ± SD
Control	0.006 ± 0.001
Low Dose Pitavastatin	0.006 ± 0.001
High Dose Pitavastatin	0.006 ± 0.001
TNF-*α*	0.190 ± 0.002
Low Dose Pitavastatin *+ *TNF-*α*	0.358 ± 0.033
High Dose Pitavastatin *+ *TNF-*α*	0.514 ± 0.044
DMSO	0.010 ± 0.000

ICAM-1, intercellular adhesion molecule-1; TNF-*α*, tumor necrosis factor-*α*; DMSO, dimethyl sulfoxide; SD, standard deviation.

**Table 2:** Results of NF-*κ*B mRNA expression levels of experimental group.

EXPERIMENT GROUP NAME	NF-*κ*B
	Mean ± SD
Control	0.005 ± 0.001
Low Dose Pitavastatin	0.003 ± 0.000
High Dose Pitavastatin	0.004 ± 0.000
TNF-*α*	0.011 ± 0.000
Low Dose Pitavastatin *+ *TNF-*α*	0.011 ± 0.001
High Dose Pitavastatin *+ *TNF-*α*	0.011 ± 0.002
DMSO	0.005 ± 0.001

NF-*κ*B, nuclear factor-kappa B; TNF-*α*, tumor necrosis factor-*α*; DMSO. dimethyl sulfoxide; SD, standard deviation.

**Table 3:** Absolute pixel density results for ICAM-1.

EXPERIMENT GROUP NAME	ICAM-1
	Mean ± SD
Control	1.8 ± 0.1
Low Dose Pitavastatin	1.9 ± 0.3
High Dose Pitavastatin	2.4 ± 0.3
TNF-*α*	8.5 ± 1.5
Low Dose Pitavastatin *+ *TNF-*α*	7.9 ± 4.5
High Dose Pitavastatin *+ *TNF-*α*	8.7 ± 4.5
DMSO	2.1 ± 0.4

ICAM-1, intercellular adhesion molecule-1; TNF-*α*, tumor necrosis factor-*α*; DMSO, dimethyl sulfoxide; SD, standard deviation.

**Table 4:** Absolute pixel density results for NF-*κ*B.

EXPERIMENT GROUP NAME	NF-*κ*B
	Mean ± SD
Control	1.31 ± 0.23
Low Dose Pitavastatin	1.28 ± 0.31
High Dose Pitavastatin	1.42 ± 0.26
TNF-*α*	2.83 ± 0.78
Low Dose Pitavastatin *+* TNF-*α*	2.18 ± 1.23
High Dose Pitavastatin *+* TNF-*α*	1.78 ± 1.24
DMSO	1.38 ± 0.34

NF-*κ*B, nuclear factor-kappa B; TNF-*α*, tumor necrosis factor-*α*; DMSO, dimethyl sulfoxide; SD, standard deviation.

**Table 5:** Comparison of ICAM-1 mrna expression levels between groups.

Comparison Groups	P Value
Control *x *Low Dose Pitavastatin	0.398
Control *x *High Dose Pitavastatin	0.432
Control *x* TNF-*α*	0.001
Control *x* Low Dose Pitavastatin *+* TNF-*α*	0.001
Control *x* High Dose Pitavastatin *+* TNF-*α*	0.001
Control *x * DMSO	0.001
Low Dose Pitavastatin* x *High Dose Pitavastatin	1.000
Low Dose Pitavastatin* x *TNF-*α*	0.001
Low Dose Pitavastatin* x *Low Dose Pitavastatin *+* TNF-*α*	0.001
Low Dose Pitavastatin* x * High Dose Pitavastatin *+* TNF-*α*	0.001
Low Dose Pitavastatin* x * DMSO	0.001
High Dose Pitavastatin *x *TNF-*α*	0.002
High Dose Pitavastatin* x *Low Dose Pitavastatin *+* TNF-*α*	0.002
High Dose Pitavastatin* x *High Dose Pitavastatin *+* TNF-*α*	0.002
High Dose Pitavastatin* x * DMSO	0.002
Low Dose Pitavastatin *+* TNF-*αx *TNF-*α*	0.001
Low Dose Pitavastatin *+* TNF-*αx *High Dose Pitavastatin *+* TNF-*α*	0.001
Low Dose Pitavastatin *+* TNF-*αx * DMSO	0.001
High Dose Pitavastatin *+* TNF-*αx *TNF-*α*	0.001
High Dose Pitavastatin *+* TNF-*αx * DMSO	0.001
TNF-*α x * DMSO	0.001

ICAM-1, intercellular adhesion molecule-1; TNF-*α*, tumor necrosis factor-*α*; DMSO, dimethyl sulfoxide.

**Table 6:** Comparison of NF-*κ*B mRNA expression levels between groups.

Comparison Groups	P Value
Control *x* Low Dose Pitavastatin	0.001
Control *x *High Dose Pitavastatin	0.002
Control *x* TNF-*α*	0.001
Control *x* Low Dose Pitavastatin *+* TNF-*α*	0.001
Control *x* High Dose Pitavastatin *+* TNF-*α*	0.001
Control *x * DMSO	1.000
Low Dose Pitavastatin* x *High Dose Pitavastatin	0.002
Low Dose Pitavastatin* x *TNF-*α*	0.001
Low Dose Pitavastatin* x *Low Dose Pitavastatin *+* TNF-*α*	0.001
Low Dose Pitavastatin* x * High Dose Pitavastatin *+* TNF-*α*	0.001
Low Dose Pitavastatin* x * DMSO	0.001
High Dose Pitavastatin *x *TNF-*α*	0.002
High Dose Pitavastatin* x *Low Dose Pitavastatin *+* TNF-*α*	0.002
High Dose Pitavastatin* x *High Dose Pitavastatin *+* TNF-*α*	0.002
High Dose Pitavastatin* x * DMSO	0.002
Low Dose Pitavastatin *+* TNF-*αx *TNF-*α*	0.205
Low Dose Pitavastatin *+* TNF-*αx *High Dose Pitavastatin *+* TNF-*α*	0.673
Low Dose Pitavastatin *+* TNF-*αx * DMSO	0.001
High Dose Pitavastatin *+* TNF-*αx *TNF-*α*	0.205
High Dose Pitavastatin *+* TNF-*αx * DMSO	0.001
TNF-*α x * DMSO	0.001

NF-*κ*B, nuclear factor-kappa B; TNF-*α*, tumor necrosis factor-*α*; DMSO, dimethyl sulfoxide.

**Table 7:** Comparison of ICAM-1 absolute pixel density between experimental groups.

Comparison Groups	P Value
Control *x* Low Dose Pitavastatin	0.133
Control *x *High Dose Pitavastatin	0.006
Control *x* TNF-*α*	0.004
Control *x* Low Dose Pitavastatin *+* TNF-*α*	0.001
Control *x* High Dose Pitavastatin *+* TNF-*α*	0.001
Control *x * DMSO	0.032
Low Dose Pitavastatin* x *High Dose Pitavastatin	0.028
Low Dose Pitavastatin* x *TNF-*α*	0.003
Low Dose Pitavastatin* x *Low Dose Pitavastatin *+* TNF-*α*	0.001
Low Dose Pitavastatin* x * High Dose Pitavastatin *+* TNF-*α*	0.001
Low Dose Pitavastatin* x * DMSO	0.565
High Dose Pitavastatin *x *TNF-*α*	0.006
High Dose Pitavastatin* x *Low Dose Pitavastatin *+* TNF-*α*	0.002
High Dose Pitavastatin* x *High Dose Pitavastatin *+* TNF-*α*	0.003
High Dose Pitavastatin* x * DMSO	0.123
Low Dose Pitavastatin *+* TNF-*αx *TNF-*α*	0.386
Low Dose Pitavastatin *+* TNF-*αx *High Dose Pitavastatin *+* TNF-*α*	0.624
Low Dose Pitavastatin *+* TNF-*αx * DMSO	0.001
High Dose Pitavastatin *+* TNF-*αx *TNF-*α*	0.814
High Dose Pitavastatin *+* TNF-*αx * DMSO	0.001
TNF-*α x * DMSO	0.003

ICAM-1, intercellular adhesion molecule-1; TNF-*α*, tumor necrosis factor-*α*; DMSO, dimethyl sulfoxide.

**Table 8:** Comparison of NF-*κ*B absolute pixel density between experimental groups.

Comparison Groups	P Value
Control *x* Low Dose Pitavastatin	1.000
Control *x *High Dose Pitavastatin	0.465
Control *x* TNF-*α*	0.006
Control *x* Low Dose Pitavastatin *+* TNF-*α*	0.034
Control *x* High Dose Pitavastatin *+* TNF-*α*	0.948
Control *x * DMSO	0.568
Low Dose Pitavastatin* x *High Dose Pitavastatin	0.361
Low Dose Pitavastatin* x *TNF-*α*	0.004
Low Dose Pitavastatin* x *Low Dose Pitavastatin *+* TNF-*α*	0.077
Low Dose Pitavastatin* x * High Dose Pitavastatin *+* TNF-*α*	0.796
Low Dose Pitavastatin* x * DMSO	0.568
High Dose Pitavastatin *x *TNF-*α*	0.011
High Dose Pitavastatin* x *Low Dose Pitavastatin *+* TNF-*α*	0.285
High Dose Pitavastatin* x *High Dose Pitavastatin *+* TNF-*α*	0.558
High Dose Pitavastatin* x * DMSO	0.808
Low Dose Pitavastatin *+* TNF-*αx *TNF-*α*	0.099
Low Dose Pitavastatin *+* TNF-*αx *High Dose Pitavastatin *+* TNF-*α*	0.123
Low Dose Pitavastatin *+* TNF-*αx * DMSO	0.153
High Dose Pitavastatin *+* TNF-*αx *TNF-*α*	0.039
High Dose Pitavastatin *+* TNF-*αx * DMSO	0.862
TNF-*α x * DMSO	0.004

NF-*κ*B, nuclear factor-kappa B; TNF-*α*, tumor necrosis factor-*α*; DMSO, dimethyl sulfoxide.

**Table 9:** Results of LDH levels of experimental groups.

EXPERIMENT GROUP NAME	LDH
	Mean ± SD
Control	0.00 ± 0.00
Low Dose Pitavastatin	2.37 ± 9.53
High Dose Pitavastatin	10.28 ± 7.54
TNF-*α*	-4.63 ± 4.78
Low Dose Pitavastatin *+* TNF-*α*	-3.12 ± 5.65
High Dose Pitavastatin *+* TNF-*α*	11.98 ± 7.89
DMSO	10.57 ± 31.74

LDH, lactate dehydrogenase; NF-*κ*B, nuclear factor-kappa B; TNF-*α*, tumor necrosis factor-*α*; DMSO, dimethyl sulfoxide; SD, standard deviation.

**Table 10:** Comparison of experimental groups in terms of LDH levels.

Comparison Groups	P Value
Control *x* Low Dose Pitavastatin	1.000
Control *x *High Dose Pitavastatin	0.053
Control *x* TNF-*α*	0.053
Control *x* Low Dose Pitavastatin *+* TNF-*α*	0.053
Control *x* High Dose Pitavastatin *+* TNF-*α*	0.053
Control *x * DMSO	0.052
Low Dose Pitavastatin* x *High Dose Pitavastatin	0.128
Low Dose Pitavastatin* x *TNF-*α*	0.109
Low Dose Pitavastatin* x *Low Dose Pitavastatin *+* TNF-*α*	0.259
Low Dose Pitavastatin* x * High Dose Pitavastatin *+* TNF-*α*	0.078
Low Dose Pitavastatin* x * DMSO	0.336
High Dose Pitavastatin *x *TNF-*α*	0.010
High Dose Pitavastatin* x *Low Dose Pitavastatin *+* TNF-*α*	0.025
High Dose Pitavastatin* x *High Dose Pitavastatin *+* TNF-*α*	1.000
High Dose Pitavastatin* x * DMSO	0.748
Low Dose Pitavastatin *+* TNF-*αx *TNF-*α*	0.520
Low Dose Pitavastatin *+* TNF-*αx *High Dose Pitavastatin *+* TNF-*α*	0.016
Low Dose Pitavastatin *+* TNF-*αx * DMSO	0.109
High Dose Pitavastatin *+* TNF-*αx *TNF-*α*	0.010
High Dose Pitavastatin *+* TNF-*αx * DMSO	0.748
TNF-*α x * DMSO	0.748

LDH, lactate dehydrogenase; TNF-*α*, tumor necrosis factor-*α*; DMSO, dimethyl sulfoxide.

## Data Availability

The data used to support the findings of this study are available from the corresponding author upon request.
